# 黄连素在A549细胞中对顺铂抗肿瘤作用的影响及其机制

**DOI:** 10.3779/j.issn.1009-3419.2015.08.03

**Published:** 2015-08-20

**Authors:** 国君 蒋, 利 李, 小祥 吴, 淑英 董, 旭辉 童

**Affiliations:** 233030 蚌埠，蚌埠医学院药学系药理学教研室，安徽省生化药物工程技术研究中心 Faculty of Pharmacy, Bengbu Medical College/Anhui Engineering Technology Research Center of Biochemical Pharmaceuticals, Bengbu 233030, China

**Keywords:** 黄连素, 缝隙连接, Cx43, Berberine, Gap junction, Connexin 43

## Abstract

**背景与目的:**

以顺铂为基础的化疗方案是晚期非小细胞肺癌的一线化疗方案，但是由于顺铂的不良反应严重及耐药性的产生均限制了它的临床应用，本研究采用联合用药的方式观察黄连素对顺铂抗肿瘤作用的影响，并探讨其可能机制。

**方法:**

分别观察黄连素对肺腺癌细胞A549细胞中总Cx43蛋白、细胞膜Cx43蛋白的表达以及细胞缝隙连接功能的改变，通过标准细胞集落克隆实验观察黄连素对顺铂细胞毒性的影响；并观察PKC激酶的表达。

**结果:**

黄连素在0 μM-10 μM浓度范围内对细胞无毒性，通过增加细胞内总Cx43蛋白和胞膜Cx43蛋白的表达而增强细胞缝隙连接功能；这种作用与PKC的活性被抑制相关，抑制PKC活性可以进一步增加顺铂对A549细胞的毒性作用。

**结论:**

黄连素可通过增加A549细胞的缝隙连接功能而明显增强顺铂的细胞毒性。

## Abstract (en)

**Background and objective:**

Cisplatin is among the standard first-line chemotherapeutic agents for treating advanced non-small cell lung cancer. Unfortunately, the clinical application cisplatin is restricted because it induces serious adverse reaction. The aim of this study is to investigate the influence and probable mechanism of berberine on cisplatin antineoplastic effect on lung cancer A549 cells.

**Methods:**

The total Cx43 protein amount, localization of Cx43 on cell membrane, and gap junction function were observed after the A549 cells were pretreated with berberine. The influence of berberine on the antitumor action of cisplatin was detected by standard colony-forming assay. Protein kinase C (PKC) protein, which regulates the gap junction, was subsequently determined.

**Results:**

Berberine did not affect cell survival at concentrations of 0 μM to 10 μM in the A549 cells. The gap junction function between the cells was enhanced through increased Cx43 protein expression and localization of Cx43 on the membrane after berberine treatment. This effect was associated with the PKC activity. The cisplatin-induced inhibition of colony growth was enhanced when berberine was combined with cisplatin.

**Conclusion:**

Berberine can obviously increase the antitumor effect of cisplatin by enhancing the function of the gap junction possibly in A549 cells.

肺癌是严重威胁人类健康的恶性肿瘤之一，以顺铂为基础的联合化疗是常见的晚期肺癌化疗方案，但是连续、大量使用不仅会出现严重的不良反应，还会使肿瘤细胞对顺铂敏感性降低，从而导致化疗失败。因此寻找增强肿瘤细胞对顺铂敏感性的方法对于逆转细胞耐药、扩大其临床应用至关重要。

缝隙连接（gap junction, GJ）是细胞间物质交换的重要蛋白通道，由连接蛋白（connexin, Cx）组成，广泛存在于实质性脏器中，如肺脏、肝脏等。近年来研究^[1]^显示GJ在肿瘤的发生、发展及转移过程中起到非常重要的作用，且本课题组前期研究^[2-4]^表明，通过采用GJ功能工具药物调控细胞GJ功能能够增加铂类药物的抗肿瘤活性。

黄连素（berberine, BBR）又名小檗碱，是从中国传统中药黄连的根、茎中提炼出的主要成分，广泛用于治疗感染性腹泻、细菌性痢疾等感染性肠道疾病。Liu等^[5]^证明了黄连素可以通过调控肿瘤细胞GJ功能进而增强X射线诱导肿瘤细胞凋亡的作用，但是具体机理不明。因此本实验通过联合应用黄连素和顺铂，观察他们对肺癌细胞的杀伤作用，并对相关机制进行探讨。

## 1 材料与方法

### 1.1 材料

肺癌细胞A549购自于ATCC公司，由蚌埠医学院药理实验室冻存。顺铂、黄连素、GF109203X、胰蛋白酶、MTT、二甲基亚砜（DMSO）均为美国Sigma公司产品；1640培养基、新生牛血清、荧光染料calcein-AM为美国Gibco公司产品；ECL发光试剂盒购于Invitrogen公司。Cx43单克隆抗体和PKC单克隆抗体均为Sigma公司产品，羊抗鼠β-actin单克隆抗体、山羊抗小鼠IgG抗体、山羊抗兔IgG抗体均购自Proteintech公司。其他常用试剂均为国产分析纯级。

### 1.2 方法

肺癌细胞A549采用1640培养基，含有10%（V/V）新生牛血清、100 U/mL青霉素和100 mg·L^-1^链霉素，置于37 ℃、含体积分数5% CO_2_以及饱和湿度的细胞培养箱中培养。细胞常规培养于培养瓶中，0.25%胰蛋白酶溶液消化传代，一周传代2次-3次，传代比例约为1:3。细胞接种荧光示踪法^[6]^测定A549细胞间GJ功能。本实验将A549细胞与荧光指示剂calcine-AM共同孵育，使calcine-AM进入细胞。该细胞称为“供体细胞”（donor cell）。将“供体细胞”接种到表达有相同Cx，已生长融合的A549细胞（“接受细胞”，receiver cells）上，培养4 h。待形成稳定的GJ后，小分子的calcine（发绿色荧光）可以通过GJ进入相邻的“接受细胞”，用荧光显微镜观察，记录GJ荧光传递功能。一个“供体细胞”周围含有calcine的“接受细胞”数目多少作为GJ功能指标。采用0.1 μM、1 μM、10 μM的黄连素分别预处理细胞24 h后，弃原培养基，1×PBS洗1次，加入“供体细胞”后孵育4 h后进行拍照统计。

### 1.3 标准细胞集落克隆实验

本研究采用Glazer报道的“标准细胞集落形成分析法”^[7]^，测定顺铂对培养细胞的细胞集落（克隆）形成的影响。本实验获得生长融合的细胞（以6×10^5^ cells/mL接种，细胞之间紧密接触，有条件形成GJ）及生长未融合细胞（以1, 000 cells/mL接种，细胞之间无条件形成GJ），首先采用10 μM黄连素预处理A549细胞24 h后，弃原培养基，再加入5 μM顺铂处理细胞1 h，测定细胞7天的集落形成率；比较生长融合细胞与生长未融合细胞的顺铂毒性大小，为了避免不同生长期的细胞对顺铂敏感性的差异对实验结果的影响，细胞在加入药物前24 h用无血清培养基培养，使所有细胞均同步在G_1_期。

### 1.4 Western blot检测A549细胞内Cx43及PKC蛋白表达

黄连素（0.1 μM、1 μM、10 μM）分别作用于细胞24 h后，冰上收集细胞，裂解30 min，提取细胞总蛋白，BCA蛋白定量法（参照试剂盒说明书操作）测各组蛋白浓度，用细胞裂解液将各组蛋白稀释至相同浓度，与2×上样缓冲液1:1混合，100 ℃煮沸5 min使蛋白变性。经SDS-PAGE电泳（10%分离胶）分离后电转印至PVDF膜上。脱脂牛奶室温封闭；Cx43一抗：1:4, 000稀释，PKC一抗：1:1, 000稀释，4℃孵育过夜；TPBS洗涤3次×15 min；山羊抗小鼠IgG抗体1:4, 000稀释，山羊抗兔IgG二抗1:5, 000稀释室温孵育2 h；TPBS洗涤3次×15 min，0.01 M PBS洗涤1次×15 min；ECL发光试剂盒显影曝光。Bio-Rad凝胶成像系统采集图像，Bio Imaging System（Gene Genius）对胶片进行灰度值扫描分析。以β-actin为内参对照，蛋白表达强度以Cx43蛋白表达灰度值与β-actin灰度值的比值表示^[8, 9]^。

### 1.5 统计学方法

实验结果使用SPSS 13.0软件进行分析。数据资料以Mean±SD表示，整体比较采用*ANOVA*，组间比较*P* < 0.05为差异有统计学意义。采用*q*检验，统计图表采用Sigma Plot 12.0绘制。

## 2 结果

### 2.1 黄连素对A549细胞生长的抑制作用

本实验采用不同浓度的黄连素（2.5 μM、5.0 μM、10.0 μM、20.0 μM、40.0 μM、80.0 μM）预处理A549细胞24 h，观察药物对细胞的生长抑制作用。实验结果表明，黄连素在0 μM-10 μM的浓度范围对A549细胞无毒性，10 μM的黄连素刺激细胞24 h后，细胞存活率为（95.24±4.19）%，仍高于95%。而在黄连素浓度高于10 μM时，随着药物浓度的增加，黄连素对A549细胞的生长抑制作用明显增加，且各药物浓度组对A549细胞的存活率与空白对照组比较，差异均具有统计学意义（*P*=0.049, 9, *P*=0.110, 9, *P*=0.000, 5, *P* < 0.001, *P* < 0.001, *P* < 0.001）（图 1）。

**1:**
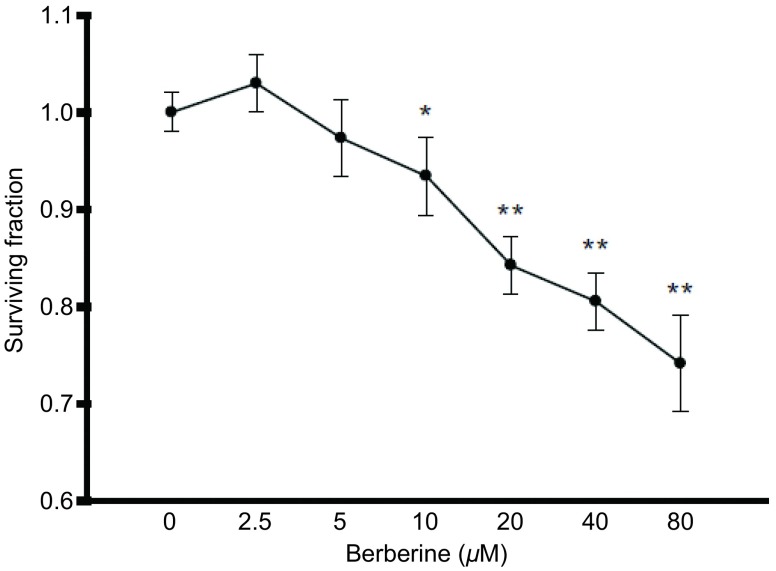
黄连素对A549细胞存活率的影响（*n*=3）。A549细胞用2.5 *μ*M-80 *μ*M黄连素处理24 h.与对照组相比：^*^P < 0.05，^**^P < 0.01。 Cytotoxicity of berberine on A549 cells for 24 h (*n*=3). A549 cells were exposed to 2.5 *μ*M-80 *μ*M berberine for 24 h. ^*^P < 0.05, ^**^P < 0.01, *vs* control group.

### 2.2 黄连素对A549细胞缝隙连接功能的影响

本实验采用不同浓度黄连素（0 μM、0.1 μM、1 μM、10 μM）预处理A549细胞24 h，细胞接种荧光示踪法实验结果表明：黄连素在0 μM-10 μM范围内可以增强细胞间的荧光传递，与空白对照组相比，黄连素预处理后的细胞间荧光传递功能分别增加了33.3%（*P*=0.002, 3）、67.0%（*P* < 0.001）、160.0%（*P* < 0.001）（图 2）。

**2:**
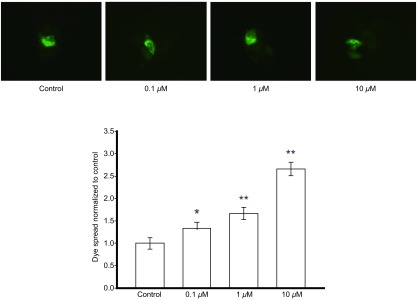
黄连素对A549细胞GJ功能的影响（×400, *n*=3）A549细胞用0 *μ*M-10 *μ*M黄连素处理24 h，对照组为空白培养基。与对照组相比：^*^P < 0.05, ^**^P < 0.01。 Effects of berberine on dye spread in A549 cells (×400, *n*=3). A549 cells were exposed to 0 *μ*M-10 *μ*M berberine for 24 h. ^*^P < 0.05, ^**^P < 0.01, *vs* control group.

### 2.3 黄连素对顺铂细胞毒性的影响

本实验分别在有GJ形成的细胞（生长融合）和无GJ形成的细胞（生长未融合）的条件下，采用“标准集落形成法”观察10 μM黄连素（从图 1结果可知，在该浓度下，黄连素本身对细胞生长无影响）对顺铂细胞毒性的影响。黄连素预处理A549细胞24 h后，再加入5 μM顺铂处理细胞1 h，观察顺铂的细胞毒性变化。

实验结果显示，在生长融合的细胞中（有GJ形成），顺铂单用组的细胞集落形成率为（0.43±0.03）%，低于生长未融合的细胞（无GJ形成）（0.53±0.05）%。在生长融合的细胞中，用黄连素（10 μM）增强细胞GJ功能，细胞集落形成率为（0.27±0.04）%，与单用顺铂组相比降低（*P*=0.002, 3）。而在生长未融合的细胞中，无GJ形成，与顺铂组相比，用黄连素预处理细胞，不影响顺铂处理后的细胞集落形成（*P*=0.067, 2）。结果表明，在有GJ形成的细胞，黄连素能显著增加顺铂的细胞毒性，而在无GJ形成的细胞，黄连素对顺铂的细胞毒性无显著影响（图 3）。

**3:**
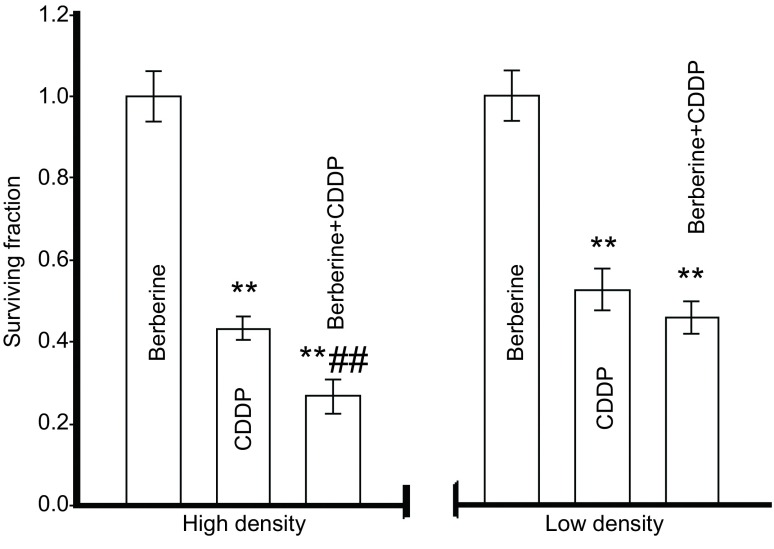
黄连素对顺铂细胞毒性的影响（*n*=3）。在高低密度情况下，检测10 *μ*M黄连素预处理细胞24 h，再联合使用5 *μ*M顺铂后A549细胞存活率。与黄连素组相比：^**^P < 0.01，与顺铂组相比：^##^*P* < 0.01。 Effects of berberine on the cytotoxicity of cisplatin in A549 cells (*n*=3). Surviving fraction of A549 cells by 5 *μ*M cisplatin for 24 h at high and low cell density and pretreated with 10 *μ*M berberine. ^**^P < 0.01, vs berberine group; ^##^*P* < 0.01, *vs* cisplatin group.

### 2.4 黄连素对A549细胞Cx43蛋白表达的影响

本实验采用不同浓度黄连素（0 μM、0.1 μM、1 μM、10 μM）分别预处理A549细胞24 h，细胞中Cx43蛋白的表达水平增加。实验结果表明，黄连素增强A549细胞GJ功能与增加了细胞内Cx43蛋白表达有关（图 4）。

**4:**
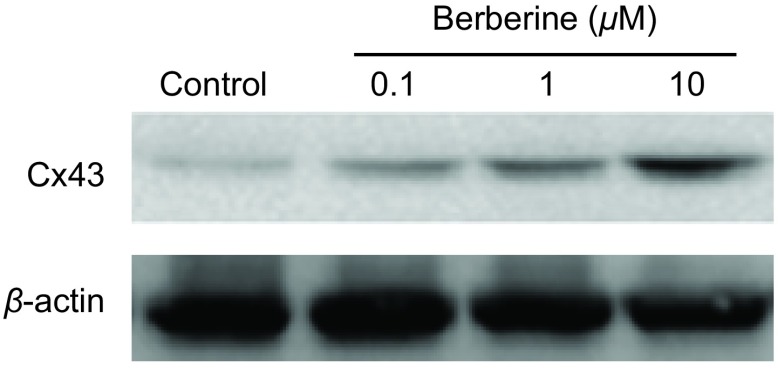
黄连素对A549细胞中Cx43蛋白表达的影响（*n*=3）。A549细胞用0 *μ*M-10 *μ*M黄连素处理24 h，对照组为空白培养基。 Effects of berberine on Cx43 expression in A549 cells determined by Western blot (*n*=3). A549 cells were exposed to 0 *μ*M-10 *μ*M berberine for 24 h

### 2.5 黄连素对A549细胞膜Cx43蛋白表达的影响

由于连接蛋白必须被转运至细胞膜，与相邻细胞形成GJ才能够允许物质进行交换。因此本实验采用细胞免疫荧光法观察了黄连素对A549细胞膜表面Cx43蛋白表达的影响。实验结果显示，0 μM-10 μM黄连素预处理A549细胞24 h，可以提高细胞膜Cx43蛋白表达水平，且随着药物浓度的增加，胞膜Cx43蛋白表达越多（图 5）。这提示，黄连素在0 μM-10 μM的浓度范围内是通过增加细胞膜Cx43蛋白的表达进而增强了细胞的GJ功能。

**5:**
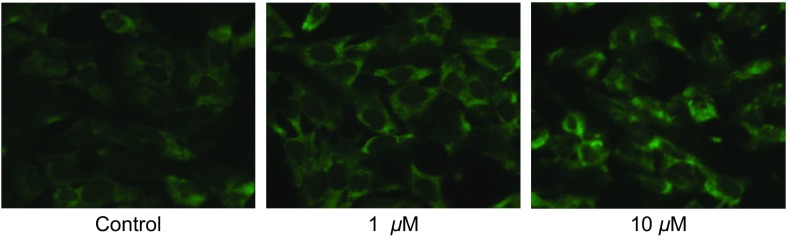
黄连素对A549细胞膜Cx43蛋白表达的影响（*n*=3）。A549细胞用0 *μ*M-10 *μ*M黄连素处理24 h，对照组为空白培养基。 Effects of berberine on Cx43 expression on the surface of A549 cells (*n*=3). A549 cells were exposed to 0 *μ*M-10 *μ*M berberine for 24 h.

### 2.6 黄连素对A549细胞中PKC蛋白表达的影响

采用不同浓度黄连素（0 μM、0.1 μM、1 μM、10 μM）预处理A549细胞24 h，细胞中PKC蛋白的表达水平降低。该实验结果提示：黄连素增加Cx43蛋白表达可能与PKC的活性被抑制有关（图 6）。

**6:**
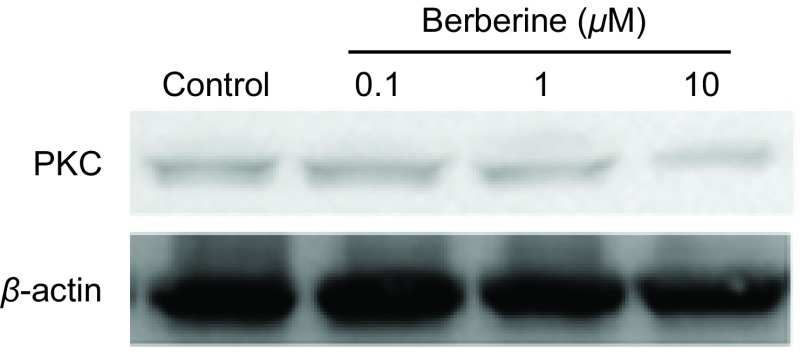
黄连素对A549细胞中PKC蛋白表达的影响（*n*=3）。A549细胞用0 *μ*M-10 *μ*M黄连素处理24 h，对照组为空白培养基。 Effects of berberine on PKC expression in A549 cells (*n*=3)(control: DMSO). A549 cells were exposed to 0 *μ*M-10 *μ*M berberine for 24 h.

### 2.7 PKC抑制剂对细胞Cx43蛋白表达的影响

为了进一步证明PKC的活性可以直接影响到细胞Cx43蛋白的表达，本实验采用PKC特异性的抑制剂GF109203X 8 μM作用于A549细胞1 h，观察对Cx43蛋白表达的影响。实验结果显示：GF109203X在抑制PKC活性的同时，可以显著增加Cx43的表达（图 7）。这表明：PKC的活性可以调控细胞Cx43蛋白。本实验证明了黄连素就是通过抑制PKC的活性而增强了Cx43蛋白的表达和细胞GJ功能，进而增加顺铂的抗肿瘤作用。

**7:**
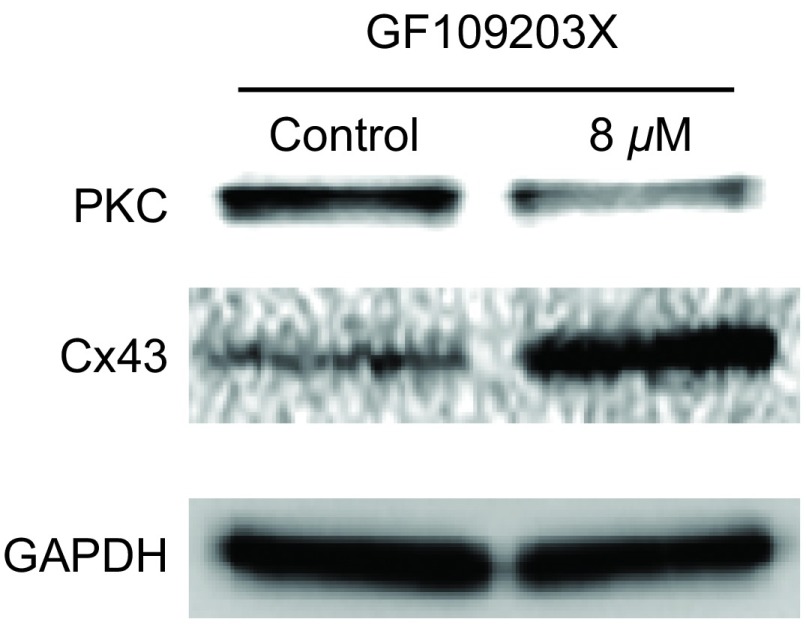
GF109203X对A549细胞中Cx43蛋白表达的影响（*n*=3）。A549细胞用8 *μ*M GF109203X处理24 h，对照组为空白培养基。 Effects of GF109203X on Cx43 expression in A549 cells (*n*=3)(control: DMSO). A549 cells were exposed to 8 *μ*M GF109203X for 24 h.

## 3 讨论

GJ是细胞间进行物质交流的重要连接通道，由特殊的通道蛋白——Cx组成^[10]^。由GJ介导的细胞间信号转导对于细胞正常生理功能及机体内环境稳定至关重要。

本研究在肺癌细胞A549中研究了黄连素对顺铂细胞毒性的影响，并探讨可能机制。本研究首先采用细胞接种荧光示踪法观察黄连素对A549细胞的GJ功能影响，结果显示1 μM-10 μM黄连素(不影响细胞生长)可以增强细胞的GJ功能；接着采用高低密度接种法接种细胞，在高密度接种细胞时（有条件形成GJ），黄连素可以明显增强顺铂的细胞毒性，而在低密度接种细胞时（无条件形成GJ），黄连素不影响顺铂的细胞毒性。这表明，黄连素通过增强A549细胞GJ功能而增加顺铂的抗肿瘤作用。

药物主要通过两种方法影响细胞的GJ功能：直接改变缝隙连接通道的通透性^[11]^和调控细胞中Cx的表达及转运^[12]^。肺腺癌细胞A549中表达最多的连接蛋白为Cx43^[13]^，于是本实验观察了黄连素对A549细胞内Cx43总蛋白表达的影响，结果显示在一定浓度范围内，黄连素可以增加细胞中Cx43蛋白的表达。这提示：黄连素就是通过增加细胞中Cx43蛋白的表达水平而增强细胞GJ功能。

Cx43从转录、翻译到转运至胞膜的过程中受到各种因素的调控，翻译后调控^[14]^中的蛋白磷酸化^[15]^已经成为当今研究热点。Cx43蛋白磷酸化的状态不同程度地影响了自身的合成及细胞的缝隙连接通讯，进而影响机体生理或者病理过程。Cx43的磷酸化受到多种激酶途径的调节，如Liao等^[16]^在新生大鼠的心肌细胞中观察到PKC的活性被溶血磷脂胆碱诱导后可以引起Cx43的丝氨酸磷酸化。于是我们推测黄连素在A549细胞中可能是通过抑制PKC的活性而阻碍了Cx43的磷酸化。本研究结果也表明黄连素可以抑制A549细胞PKC蛋白的表达。为了证明在肺癌细胞A549中PKC的抑制可以增加Cx43总蛋白的表达，实验采用PKC抑制剂GF109203X预处理A549细胞，结果显示GF109203X预处理的细胞Cx43表达增加。

PKC是一种由第二信使激活的磷脂依赖性蛋白激酶，它在信号转导和肿瘤细胞增殖中起关键作用。有研究^[17]^表明，PKC介导的Cx43磷酸化可以减少GJ的装配、下调GJIC，并且缩短Cx43的半衰期；也有研究^[18]^证明使用PKC抑制剂可以增加细胞GJ的电偶联，而与细胞Cx43蛋白的分布及蛋白磷酸化的状态无关。这表明，PKC影响细胞GJ功能是否与调控Cx43相关具有组织差异性。本研究证明了，在肺腺癌细胞A549中PKC对细胞GJ功能的调控是通过影响Cx43的表达而实现的。

本研究为临床肺癌晚期治疗用药选择提供了参考依据，为顺铂在临床的扩大应用提供了新的方向，GJ的调控可能会成为未来肺癌治疗的新靶点。
